# Graft function and health status in renal transplant recipients hospitalized for COVID-19: a single center case series

**DOI:** 10.1007/s40620-022-01451-5

**Published:** 2022-09-05

**Authors:** Benjamin Giszas, Johannes Ruhe, Mandy Schlosser, Philipp Alexander Reuken, Ulrike John-Kroegel, Andreas Stallmach, Gunter Wolf

**Affiliations:** 1 Department of Internal Medicine IV (Gastroenterology, Hepatology, and Infectious Diseases), Jena University Hospital/Friedrich-Schiller-University Jena, Am Klinikum 1, 07747 Jena, Germany; 2 Department of Internal Medicine III (Nephrology, Endocrinology, and Rheumatology), Jena University Hospital/Friedrich-Schiller-University Jena, Am Klinikum 1, 07747 Jena, Germany; 3 Section of Pediatric Nephrology, Department of Pediatrics, Jena University Hospital/Friedrich-Schiller-University Jena, Am Klinikum 1, 07747 Jena, Germany

**Keywords:** COVID-19, Renal transplant recipients, Immunosuppressive drugs, Acute kidney injury, Quality of life

Renal transplant recipients (RTRs) infected with SARS-CoV-2 are at increased risk for severe to fatal COVID-19, concomitant acute kidney injury (AKI), and persistent post-COVID symptoms [1, 2]. Given the previous results, a continuing negative impact on the health of the RTRs following COVID-19 can be expected. Therefore, this case series aimed to examine the long-term impact of COVID-19 on graft-function in RTRs hospitalized with moderate and severe disease. We also assessed mortality rates as well as the health status and prevalence of post-COVID symptoms at 90-days follow-up.

In this study, we enrolled 17 RTRs who were hospitalized with symptomatic SARS-CoV-2 infection. The average patient age was 56 years (interquartile range [IQR], 53.5–69.5 years) and included five (29%) women. Only one of the RTRs underwent transplantation from a living donor. The median time after transplantation was 6.9 (IQR 4.3–12.1) years. Sixteen (94.1%) were treated with immunosuppressive medications, including calcineurin inhibitors (CNIs), primarily cyclosporine or tacrolimus. The most common secondary conditions included hypertension (17, 100%), diabetes (11, 65%), and coronary heart disease (6, 35%); three of the RTRs (18%) presented with pre-existing chronic lung disease (Table S1). Fifteen (88.2%) ultimately required oxygen support during their hospital stay, albeit without evidence or clinical suspicion of pulmonary embolism. Two patients (12%) developed progressive hypoxia and required invasive ventilation.

While three patients (18%) succumbed to COVID-19 while in the hospital, none died during the follow-up period. Survivors were significantly younger (*p* = 0.008) and had lower serum levels of inflammatory markers, including procalcitonin (*p* = 0.017) and C-reactive protein (*p* = 0.032) and increased levels of D-dimers (*p* = 0.012) (Fig. S1). Taking into account the individual baseline serum creatinine levels, we found that 11 (65%) patients developed AKI (Stage I: 9, 82%; Stage III: 2, 18%, including one patient that required renal replacement therapy) with a median increase in serum creatinine of 0.5 (IQR 0.1–0.5) mg/dl. Seven (41%) of these patients presented with increased CNI levels (defined as a ≥ 1.5-fold increase above individual trough levels; e.g., tacrolimus levels up to 38.5 µg/l). The three patients who died while in the hospital (18%) developed both AKI and CNI overdose.

Eleven (79%) of the 14 survivors exhibited had completely recovered renal function when examined at a 90-days follow-up visit (Fig. 1A). In two cases, serum creatinine levels had increased over the pre-COVID-19 baseline by 0.4 and 0.9 mg/dl, respectively. The one patient who exhibited an increase of 1.8 mg/dl had previously been diagnosed with end-stage kidney disease and was not undergoing dialysis.

**Fig. 1:**
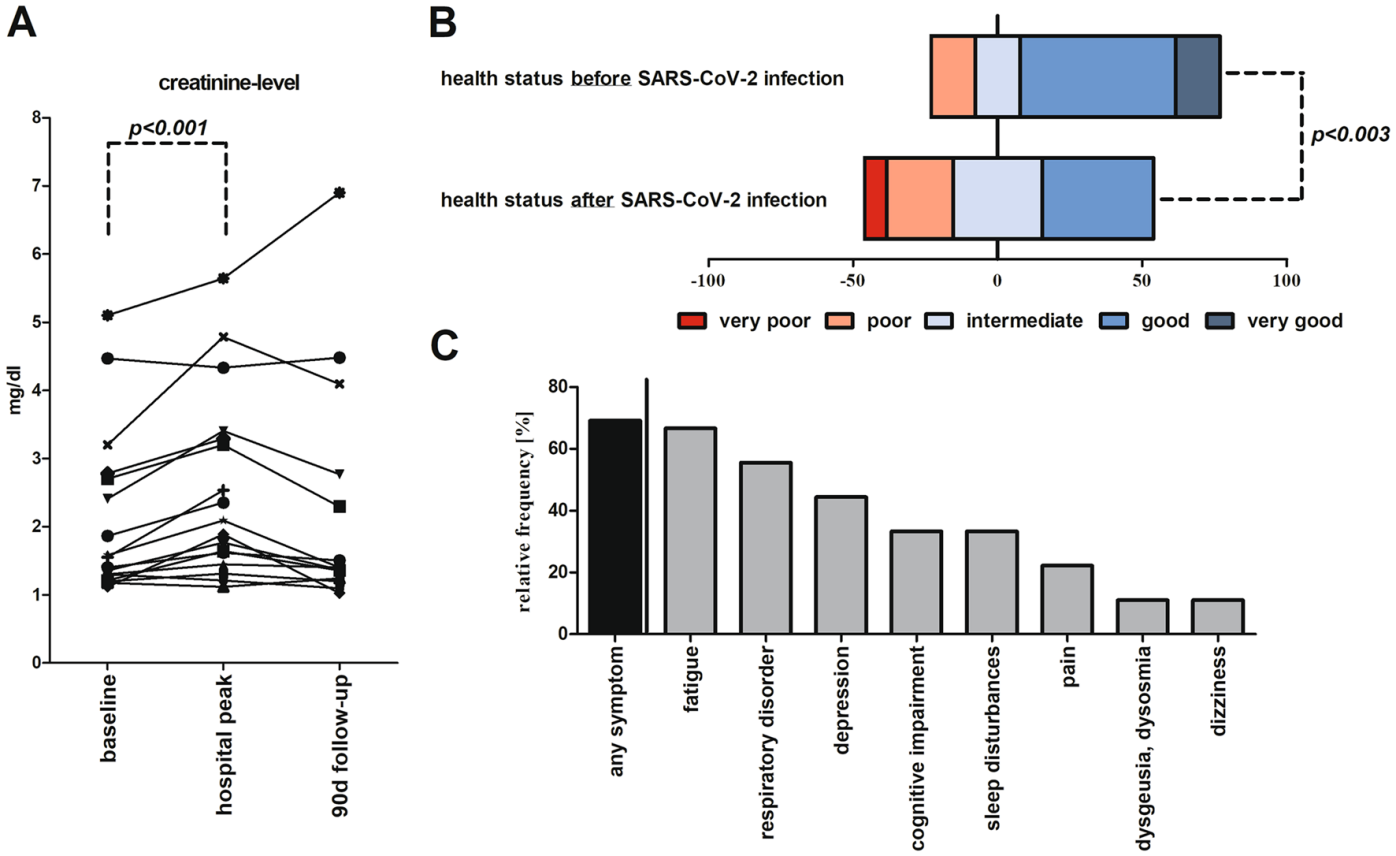
Renal status and subjective assessments of health among RTRs hospitalized for COVID-19. **A** Serum creatinine levels (baseline, peak while hospitalized, and at 90-days follow-up). **B** Self-assessed health status (Likert scale) at 90-days follow-up. Nine of the 13 patients (69%) that participated in the follow-up interview reported persistent symptoms at this time. **C** The percentage of patients reporting each symptom at 90-days follow-up

Of the 14 survivors, 13 (93%) participated in a follow-up interview designed to evaluate their current health status (Table S3). Overall, nine (69%) patients reported diminished health status at 90 days (Likert scale, Fig. 1B). The remaining four (31%) responded that their health status was unchanged, although one of these patients (8%) reported poor health status before hospitalization for COVID-19. Five patients (38%) described their current health status as good. Nine of 13 patients (69%) reported persistent symptoms at 90 days, suggesting that they may have developed post-COVID-19 condition. The most frequent complaints were fatigue (6, 67%) and dyspnea (5, 56%) (Fig. 1C).

In summary, we have reviewed and documented the outcomes of a cohort of 17 patients at high risk for severe COVID-19. The relatively high mortality (3 patients, or 18%) is consistent with findings reported in previously published studies [3]. Furthermore, our results suggested that elevated levels of therapeutic CNIs were associated with increased mortality.

Eleven of the 17 patients (65%) developed AKI; this rate is higher than the 52% previously described in the literature [1]. All three patients who died while in the hospital also developed AKI; one of these patients required renal replacement therapy. In contrast to results reported by Chauhan et al. [4], we observed elevated levels of immunosuppressive medications in seven (41%) of the 17 patients in our cohort with no apparent association with the incidence or severity of AKI. Nonetheless, we did note that all three patients who developed both AKI and elevated CNI levels died while in the hospital. These findings suggest that this combination of risk factors may be potentially hazardous.

Although AKI may lead to persistently reduced renal function, we found that serum creatinine levels measured at 90 days follow-up were generally no higher than pre-infection levels. These findings suggest that AKI and renal dysfunction that develops in RTRs in response to COVID-19 may be due to factors that are largely reversible [5]. However, we recognize that graft function will need to be monitored over a longer period (from years to decades) to exclude any and all COVID-19-associated effects.

Nearly 70% of all patients in our follow-up cohort met the criteria for post-COVID condition as defined by the World Health Organization. Furthermore, nine patients (69%) reported diminished health-related quality of life (hrQoL); this value achieved statistical significance in our single-center observational study. We anticipate ongoing although potentially only partial recovery in this cohort based on the results of a previous report in which only 48% of RTRs reported a reduced hrQoL at six months follow-up (2). Therefore, in addition to ongoing nephrological care, these patients should be provided with interdisciplinary treatment in special post-COVID outpatient clinics, particularly for those who report multiple ongoing symptoms.

In conclusion, our findings confirm that AKI is common in RTRs hospitalized for COVID-19 and that CNI-overdose appears to be directly associated with in-hospital mortality. However, our findings indicate that, among those who survive the acute infection, graft function typically stabilizes to baseline levels at 90-days follow-up. Many RTRs develop post-COVID condition with impaired hrQoL persisting as long as 3 months after moderate or severe disease. These findings emphasize the importance of providing intensified and interdisciplinary care for this vulnerable patient cohort.

## Supplementary Information

Below is the link to the electronic supplementary material.

Supplementary file1 (DOCX 115 kb)
