# Supplementary material for: Graft function and health status in renal transplant recipients hospitalized for COVID-19: a single center case series
Source: J Nephrol. 2022 Sep 5;36(3):613–5. doi: 10.1007/s40620-022-01451-5 (PMC9442566; doi:10.1007/s40620-022-01451-5)
Supplement: Supplementary file 1 — Supplementary file1 (DOCX 115 kb) [file 40620_2022_1451_MOESM1_ESM.docx]

**Supplementary Material**

Table of contents.

[Methods 2](#_Toc108771380)

[Supplemental Figure S1 - Comparison of clinical and laboratory parameters in relation to hospital survival. 4](#_Toc108771381)

[Supplemental Table 1 - Description of the study population and clinical data due to SARS-CoV-2 infection 5](#_Toc108771382)

[Supplemental Table 2 - Follow-up data 90d after SARS-CoV-2 infection 7](#_Toc108771383)

# Methods

We describe a case series of renal transplant recipients who were hospitalized via emergency admission and inpatients treated for more than 24 hours at Jena University Hospital primarily for SARS-CoV-2 infection between October 2020 and January 2022. We focused on SARS-CoV-2 first-time infections. In addition, renal function without the need for renal replacement therapy on admission was also part of our inclusion criteria.

The study was approved by the institutional ethics committee of the Friedrich Schiller University Jena (2020-1711-Daten, 2022-2630-Bef) and conducted according to the Declaration of Helsinki and Istanbul.

Patients with combined organ transplants and hospitalizations for other causes were excluded, as were nosocomial SARS-CoV-2 infections.

On admission to hospital, reverse transcription-polymerase chain reaction (RT-PCR) for SARS-CoV-2 was performed by oropharyngeal or nasal swab. The resulting cycle time (ct) value was measured to assess viral load (day 0).

Primary endpoints were mortality during hospitalization, and renal graft function and health status after 90 days of follow-up. Secondary endpoints were mortality at days 30, 60, and 90 and renal graft function during the hospital stay, respectively. Renal function is described as estimated glomerular filtration rate (eGFR) using the CKD-EPI formula and reported at G stage of CKD. During SARS-CoV-2 infection, all patients were under nephrological care. Clinical symptoms, medical history along with laboratory parameters, including CNI trough levels, were retrospectively analyzed using the electronic medical record noted at admission and in the discharge letter. CNI-overdose was assumed if the individual trough-level exceeded highest limit value by 1.5 times.

Most of the included patients were already in outpatient care at our renal transplant outpatient clinic before SARS-CoV-2 infection. Structured follow-up of SARS-CoV-2 survivors to assess renal function was performed during regular consultations and in cooperation with local nephrology centers.

A short questionnaire-based interview was used to assess health status at follow-up and to identify post-COVID condition, using a Likert-scale. The translated questionnaire can be found in the supplementary material. Exclusion criteria for this assessment were death before day 90 and communication problems. The structured interview was conducted in person or by telephone 90 days after the first RT-PCR. Thirteen patients were included. All of these patients gave written informed consent.

**Statistics**

Baseline characteristics are described by means (± standard deviation; SD) for normally distributed variables and by median values and interquartile ranges (Q1; Q3) for non-normally distributed variables. Values of categorical variables are presented as frequency distributions with percentages. Statistical analysis of each variable is based only on available values and percentages related to the total number of available values. For the analysis of differences between groups, Pearson's chi-square test and Fisher's exact test were used for categorical variables, while Mann-Whitney U test and Kruskal-Wallis test were used for numerical variables. Wilcoxon test was used for the assessment of course parameters (paired variables).

All p values were two-sided, and p < 0.05 was considered significant. All analyses were performed using IBM SPSS version 26 and GraphPad Prism6.

**Questionnaire**

1. Have you been admitted to hospital within three months after initial hospitalization due to SARS-CoV-2 infection?

O No

O Yes

- 1. What was the main reason for admission? _______________________
  2. Was it a planned/elective admission?

O Yes

O No (Emergency Admission)

- 1. Was the admission mainly associated with the renal transplant graft?

O Yes

O No

1. How would you describe your state of health before the infection with SARS-CoV-2?

O Very good

O Good

O Medium

O Poor

O Very poor

1. How would you describe your state of health 90 days after the infection with SARS-CoV-2?

O Very good

O Good

O Medium

O Poor

O Very poor

1. Did one or more of the following symptoms occur as new onset after the infection? (Tick, if applicable)

O No

O Yes

- 1. Fatigue, tiredness, reduced working capacity
  2. Sleep disturbances
  3. Pain, reduced mobility, muscular problems
  4. Shortness of breath, cough, reduced breathing capacity at physical activity
  5. Taste or smelling disorder (dysgeusia, dysosmia)
  6. Problems to concentrate or memory problems
  7. Sorrows, sadness, depression
  8. Dizziness or vertigo, tinnitus

# Supplemental Figure S1 - Comparison of clinical and laboratory parameters in relation to hospital survival.

# Supplemental Table 1 - Description of the study population and clinical data due to SARS-CoV-2 infection

| **Characteristics** | **Total case series (n = 17)** |
| --- | --- |
| Female sex; n (%) | 5 (29.4%) |
| Age, in years; median (Q1, Q3), Minimum–Maximum | 56 (53.5/69.5) 18 - 86 |
| Body mass index; median (Q1, Q3), Minimum–Maximum | 25 (22.95/28.75) 21 - 33.5 |
| Pre-existing conditions |  |
| hypertension; n (%) | 17 (100%) |
| obesity; n (%) | 3 (17.6%) |
| coronary heart failure; n (%) | 6 (35.3%) |
| diabetes mellitus; n (%),  HbA1c; median (Q1, Q3), Minimum–Maximum | 11 (64.7%),  6.9 (6.6/8.2) 5.3 - 9.1 |
| chronic lung disease; n (%) | 3 (17.6%) |
| Type of transplant |  |
| Living-donor *renal transplant* (LDRT) | 16 (94.1%) |
| Cadaver-donor *renal transplant* (CDRT) | 1 (5.9%) |
| Number of years after transplant; median (Q1, Q3), Minimum–Maximum | 6.9 (4.3/12.1) 2.3 - 17.6 |
| Immune-modulating drugs |  |
| calcineurin inhibitor (CNI) | 16 (94.1%) |
| mammalian target of rapamycin inhibitor (mTOR-I) | 1 (5.9%) |
| Number of immune-modulating drugs; median (Q1, Q3), Minimum–Maximum | 3 (2/3) 2 - 3 |
| G-stage of chronic kidney disease (CKD) |  |
| G1; n (%) | 1 (5.9 %) |
| G2; n (%) | 3 (17.6%) |
| G3; n (%) | 7 (41.2%) |
| G4; n (%) | 5 (29.4%) |
| G5; n (%) | 1 (5.9%) |
| Number of antihypertensive drugs; median (Q1, Q3), Minimum–Maximum | 4 (2/5) 1 - 7 |
| Vaccination status |  |
| no | 6 (35.3%) |
| incomplete vaccinated | 1 (5.9%) |
| vaccinated (min. 2) | 10 (58.8%) |
| Waves of SARS-CoV-2 in Germany (calendar weeks (cw)) |  |
| CW40/2020 - CW 8/2021 | 5 (29.4%) |
| CW9/2021 - CW26/2021 | 2 (11.8%) |
| CW27/2021 - CW52/2021 | 8 (47.1%) |
| CW52/2021 - CW9/2022 | 2 (11.8%) |
| Symptoms prior to hospitalization |  |
| fever | 7 (41.2%) |
| weakness | 15 (88.2%) |
| muscle pain | 3 (17.6%) |
| nausea | 4 (23.5%) |
| vomiting | 2 (11.8%) |
| diarrhea | 4 (23.5%) |
| loss of smell and taste | 1 (5.9%) |
| cough | 10 (58.8%) |
| sore throat | 2 (11.8%) |
| shortness of breath | 10 (58.8%) |
| dizziness | 7 (41.2%) |
| Number of acute Symptoms; median (Q1, Q3), Minimum–Maximum | 3 (3/4.5) 1 - 8 |
| hospital admission |  |
| Ct-value; median (Q1, Q3), Minimum–Maximum | 21.8 (17.8/23.5) 17 - 34.5 |
| Laboratory parameters |  |
| Interleukin 6 | 63.1 (33/298) 13.3 - 882 |
| Procalcitonin (PCT) | 0.48 (0.13/2.23) 0.06 - 45.4 |
| C-reactive Protein (CRP) | 106.2 (78.95/170.5) 22.7 - 251.8 |
| Ferritin | 1183.3 (557.1/2150.65) 290 - 7911.5 |
| Summarized COVID Inflammation Score (CIS); median (Q1, Q3), Minimum–Maximum | 10 (9.5/12) 5 - 13 |
| hyperinflammation (defined by CIS ≥ 10); n (%) | 13 (76.5%) |
| Acute kidney injury; n (%) | 11 (64.7%) |
| AKIN I | 9 (81.8%) |
| AKIN II | 0 (0%) |
| AKIN III | 2 (18.2%) |
| Overdose of the immune-modulating drug (defined ≥ 1.5-fold of trough level) | 7 (41.2%) |
| Radiologic aspects |  |
| Pulmonary infiltrates; n (%) | 16 (94.1%) |
| Severity of infiltrates |  |
| mild; n (%) | 3 (18.8%) |
| moderate; n (%) | 9 (56.3%) |
| severe; n (%) | 3 (18.8%) |
| Therapeutic aspects |  |
| In need of oxygen support; n (%) | 15 (88.2%) |
| Administration of neutralizing monoclonal antibodies; n (%) | 9 (52.9%) |
| Additional antibiotic therapy; n (%) | 7 (41.2%) |
| Intensive care unit (ICU) stay; n (%) | 4 (23.5%) |
| Invasive ventilation; n (%) | 3 (17.6%) |
| Renal replacement therapy; n (%) | 1 (5.9%) |
| WHO grade |  |
| 1; n (%) | - |
| 2; n (%) | - |
| 3; n (%) | 2 (11.8%) |
| 4; n (%) | 11 (64.7%) |
| 5; n (%) | 1 (5.9%) |
| 6; n (%) | - |
| 7; n (%) | - |
| 8; n (%) (defined as death by COVID-19) | 3 (17.6%) |
| hospital days; median (Q1, Q3), Minimum–Maximum | 8 (6.5/12) 4 - 15 |

# Supplemental Table 2 - Follow-up data 90d after SARS-CoV-2 infection

| **Characteristics** | **Number of patients with missing information** | **follow-up cases (n = 14)** |
| --- | --- | --- |
| Renal function |  |  |
| stable or better |  | 11 (78.6%) |
| Worsened (defined as increase of creatinine ≥ 0.3 mg/dl) |  | 3 (21.4%) |
| Number of patients with hospital admission within 90d | 1 | 4 (30.8%) |
| Admissions mainly associated with renal transplant graft |  | 2 (15.4%) |
| planned admission (n; %) |  | 2 (15.4%) |
| Post-COVID condition after 90d | 1 | 9 (69.2%) |
| Post-COVID symptoms of RTRs with post-COVID condition | 1 |  |
| Fatigue, tiredness, reduced working capacity |  | 6 (66.7%) |
| Sleep disturbances |  | 3 (33.3%) |
| Pain, reduced mobility, muscular problems |  | 2 (22.2%) |
| Shortness of breath, cough, reduced breathing capacity at physical activity |  | 5 (55.6%) |
| Taste or smell disorder (dysgeusia, dysosmia) |  | 1 (11.1%) |
| Problems concentrating or memory problems |  | 3 (33.3%) |
| Sorrow, sadness, depression |  | 4 (44.4%) |
| Dizziness or vertigo, tinnitus |  | 1 (11.1%) |
| Number of acute Symptoms; median (Q1, Q3), Minimum–Maximum |  | 3 (1/4) 1 - 5 |
| Changes in self-reported Health status | 1 |  |
| worsened |  | 9 (69.2%) |
| unchanged |  | 4 (30.8%) |
| improved |  | 0 (0%) |
| Self-reported Health status prior to COVID-19 (n; %) | 1 |  |
| Very poor |  | 0 (0%) |
| Poor |  | 2 (15.38%) |
| Medium |  | 2 (15.38%) |
| Good |  | 7 (53.85%) |
| Very good |  | 2 (15.38%) |
| Self-reported Health status after COVID-19 (n; %) | 1 |  |
| Very poor |  | 1 (7.69%) |
| Poor |  | 3 (23.07%) |
| Medium |  | 4 (30.77%) |
| Good |  | 5 (38.46%) |
| Very good |  | 0 (0%) |
